# Public Knowledge, Attitude, and Practice Toward Herpes Zoster Vaccination in Saudi Arabia

**DOI:** 10.7759/cureus.49396

**Published:** 2023-11-25

**Authors:** Lujain A Alleft, Lama S Alhosaini, Haifa M Almutlaq, Yara M Alshayea, Shahad H Alshammari​​, Manal A Aldosari, Fahad A Alateeq

**Affiliations:** 1 College of Medicine, Imam Mohammad Ibn Saud Islamic University, Riyadh, SAU; 2 Faculty of Medicine, Medical Education, and Family Medicine, Imam Mohammad Ibn Saud Islamic University, Riyadh, SAU

**Keywords:** awareness knowledge and practice, herpes zoster vaccine, shingles, vaccine, herpes zoster

## Abstract

Introduction

Herpes zoster (HZ) is a viral infection that occurs due to the reactivation of the varicella-zoster virus (VZV). Reactivation of the latent virus causes a painful dermatomal rash that is typical in HZ, which is frequently accompanied by post-herpetic neuralgia (PHN). Although HZ negatively impacts individuals’ quality of life, vaccination has been shown to reduce the incidence of HZ and PHN and reduce the severity of the disease in the event of a breakthrough. Nonetheless, several studies have shown a low level of knowledge and poor practices regarding HZ and its vaccine. However, only two studies on this issue have been conducted in the Middle East. This study aimed to assess the level of knowledge, attitudes, and practices toward HZ vaccinations among the Saudi population aged 50 years and older.

Methods

A cross-sectional observational study was conducted from December 2022 to July 2023 involving citizens aged 50 years and older in Saudi Arabia. Data were collected using an online, validated, close-ended structured questionnaire distributed through social media. Linear regression analysis was used to assess independent predictors of knowledge regarding HZ, knowledge regarding the HZ vaccine, and attitudes toward HZ. Categorical variables were dummy-coded. Binary logistic regression was used to assess factors associated with the willingness to receive the HZ vaccine.

Results

A total of 368 respondents completed the questionnaire. The mean knowledge scores (%) regarding HZ and its vaccine were 28.6% and 37.1%, respectively. While 51.6% (n = 190) claimed to be aware of the HZ vaccine, only 31.6% (n = 60) had a knowledge score of 80% or higher. Multivariate analysis showed that knowledge was positively associated with female gender, prior chickenpox infection, and higher education. Only 54.4% of the respondents were willing to get the HZ vaccine, and 28.8% were willing to pay out of pocket for the HZ vaccine.

Conclusion

The results suggest that educational campaigns on HZ and its vaccine targeting at-risk groups are required to raise awareness and increase the public’s knowledge. Additionally, healthcare personnel's recommendation of the HZ vaccine to the target population should be encouraged, as it is an important factor in vaccine acceptability.

## Introduction

Varicella-zoster virus (VZV) is an alpha herpes virus that is responsible for chickenpox and typically occurs during childhood. It is distinguished by a maculopapular, vesicular rash that develops into dried crusts during a three to seven-day period. Herpes zoster (HZ) is a viral infection that occurs due to the reactivation of VZV [1]. Reactivation of the latent virus causes a painful dermatomal rash that is typical in HZ, which is frequently accompanied by post-herpetic neuralgia (PHN) [2]. Exposure to HZ occurs either through vaccination during childhood or exposure to those with active viremia.

Most adults are VZV seropositive, putting them at risk for HZ [3]. The risk of developing HZ increases with age, with a sharp increase after 50 years of age [3]. Preventive medicine is considered the most successful strategy for safeguarding health in older populations, and immunization against the most common infectious illnesses is the preferred strategy [4]. Vaccination has been shown to reduce the incidence of HZ and PHN and reduce the severity of the disease in the event of a breakthrough. A live, attenuated HZ vaccine (Zostavax®) has been available since 2006 to prevent HZ in adults aged ≥ 50 years, although it is contraindicated in immunocompromised patients [5,6]. In 2017, the CDC approved a subunit recombinant HZ vaccine (Shingrix®) as a two-dose recombinant for individuals 50 years of age and older. In contrast to Zostavax®, immunocompromised adults 19 years and older could receive Shingrix®, which has a 90% success rate [7,8].

Without the HZ vaccination, it is predicted that 20-30% of the population and 50% of people who live to be 85 years old will contract HZ [9]. Although HZ negatively impacts individuals’ quality of life, a systematic review of 13 studies estimated that the pooled HZ vaccination willingness rate was only 55.74%. Of the included studies, only one was conducted in the United Arab Emirates (UAE) region, with a reported willingness rate of 71.9% [10].

UAE population's knowledge, attitudes, and practices (KAP) related to the HZ virus and its vaccination were assessed in a cross-sectional study, which included 420 participants [11]. Only 1.9% of those who claimed to be knowledgeable about HZ scored 80% or above. Interestingly, three-quarters of the respondents could not establish a link between HZ and chickenpox. The results suggested a below-average level of knowledge regarding HZ and its vaccination, although the attitudes were favorable [11]. Another study in the Western region of Saudi Arabia showed that the general Saudi population had a good understanding of HZ and positive attitudes toward the HZ vaccine, although poor practices were observed [12]. However, the questionnaire that was used mainly investigated knowledge of the risk factors of HZ. Thus, the current study aimed to assess the KAP related to HZ vaccinations among the Saudi population 50 years of age and older using a more comprehensive questionnaire.

## Materials and methods

Study design

A cross-sectional observational study was conducted from December 2022 to July 2023 involving citizens aged 50 years and older in Saudi Arabia. Informed consent was obtained from all participants before administering the questionnaire, and the purpose of the study was clearly explained. Data were collected from self-reported online surveys distributed through social media apps (e.g., WhatsApp) and Telegram group chats.

Study population

The study population included adults aged 50 years and older in Saudi Arabia. The sample size was 359, with a confidence level of 95%. The sample size was determined according to the Raosoft sample size calculator (Raosoft, Inc., Seattle, WA). Only adults aged 50 years and older living in Saudi Arabia were included.

Research instrument and data collection process

A close-ended structured questionnaire was adapted from previous studies conducted in Hong Kong and the UAE [11,13]. However, the questionnaire was modified to reduce the number of questions and the time burden on the respondents. The modified version included 24 close-ended questions split into four sections: demographics (seven questions), awareness regarding HZ and its vaccine (two questions), knowledge about HZ and its vaccine (nine questions), and attitudes toward the HZ vaccine (four questions). Six questions were used to assess the respondents’ knowledge regarding HZ, and three were used to assess their knowledge regarding the HZ vaccine. True/false (T/F), multiple-choice questions (MCQ), and check-box questions were used.

Calculation of knowledge and attitude scores

For the MCQ and T/F questions, only one correct answer was possible. Respondents were awarded one point if they answered correctly and zero otherwise. Similarly, they were awarded one point for each correct answer to check-box questions. One point was deducted for each incorrect response. The total knowledge score was calculated by summing the overall number of points for each respondent. The correct answers are shown in the Supplementary Table. For knowledge regarding HZ, a maximum score of 10 was possible. For knowledge regarding vaccines, a maximum score of five was possible.

Statistical analysis

Statistical analysis was performed using R version 4.3 (R Foundation for Statistical Computing, Vienna, Austria) [14]. Frequency analyses were then conducted for each variable. Inferential statistics tests were used, including the chi-square, analysis of variance (ANOVA), and Student’s t-test. A linear regression analysis was used to assess independent predictors of knowledge regarding HZ, knowledge regarding the HZ vaccine, and attitude toward HZ. Categorical variables were dummy-coded. Binary logistic regression was used to assess factors associated with the willingness to receive the HZ vaccine. A p-value less than 0.05 was deemed statistically significant.

Ethical consideration

Ethical approval for this study’s conduction and data collection were reviewed and approved by the Institutional Review Board (IRB) Committee of Al-Imam Muhammad Ibn Saud Islamic University (Project number: 462/2023).

## Results

Demographics and awareness regarding HZ and its vaccine

A total of 368 respondents completed the questionnaire. The demographic characteristics of the respondents and their awareness of HZ are shown in Table 1.

**Table 1:** Descriptive statistics and awareness regarding HZ and its vaccine HZ: herpes zoster. P-values < 0.05 are deemed statistically significant.

		Aware of HZ			Aware of the HZ vaccine		
	All, N = 368	No, N = 62	Yes, N = 306	P-value	No, N = 178	Yes, N = 190	P-value
	N (%)	N (%)	N (%)		N (%)	N (%)	
Gender				0.002			0.097
Female	244 (66.3%)	30 (12.3%)	214 (87.7%)		110 (45.1%)	134 (54.9%)	
Male	124 (33.7%)	32 (25.8%)	92 (74.2%)		68 (54.8%)	56 (45.2%)	
Age				0.005			0.288
50–55	214 (58.2%)	32 (15.0%)	182 (85.0%)		99 (46.3%)	115 (53.7%)	
56–60	97 (26.4%)	12 (12.4%)	85 (87.6%)		46 (47.4%)	51 (52.6%)	
More than 60	57 (15.5%)	18 (31.6%)	39 (68.4%)		33 (57.9%)	24 (42.1%)	
Nationality				0.584			0.026
Non-Saudi	32 (8.70%)	7 (21.9%)	25 (78.1%)		22 (68.8%)	10 (31.2%)	
Saudi	336 (91.3%)	55 (16.4%)	281 (83.6%)		156 (46.4%)	180 (53.6%)	
City of residency				0.024			0.003
Central Region	69 (18.8%)	13 (18.8%)	56 (81.2%)		37 (53.6%)	32 (46.4%)	
Eastern Region	48 (13.0%)	15 (31.2%)	33 (68.8%)		31 (64.6%)	17 (35.4%)	
Northern Region	16 (4.35%)	3 (18.8%)	13 (81.2%)		12 (75.0%)	4 (25.0%)	
Southern Region	66 (17.9%)	12 (18.2%)	54 (81.8%)		32 (48.5%)	34 (51.5%)	
Western Region	169 (45.9%)	19 (11.2%)	150 (88.8%)		66 (39.1%)	103 (60.9%)	
Employment status				0.708			0.821
Employed	189 (51.4%)	30 (15.9%)	159 (84.1%)		93 (49.2%)	96 (50.8%)	
Retired	179 (48.6%)	32 (17.9%)	147 (82.1%)		85 (47.5%)	94 (52.5%)	
Educational level				<0.001			<0.001
Illiterate	19 (5.16%)	14 (73.7%)	5 (26.3%)		18 (94.7%)	1 (5.26%)	
Elementary	14 (3.80%)	5 (35.7%)	9 (64.3%)		9 (64.3%)	5 (35.7%)	
Intermediate	26 (7.07%)	8 (30.8%)	18 (69.2%)		16 (61.5%)	10 (38.5%)	
Secondary diploma	98 (26.6%)	15 (15.3%)	83 (84.7%)		50 (51.0%)	48 (49.0%)	
Higher education	24 (6.52%)	4 (16.7%)	20 (83.3%)		8 (33.3%)	16 (66.7%)	
University degree	187 (50.8%)	16 (8.56%)	171 (91.4%)		77 (41.2%)	110 (58.8%)	
Ever had chickenpox				<0.001			<0.001
I do not know	61 (16.6%)	20 (32.8%)	41 (67.2%)		40 (65.6%)	21 (34.4%)	
No	182 (49.5%)	31 (17.0%)	151 (83.0%)		94 (51.6%)	88 (48.4%)	
Yes	125 (34.0%)	11 (8.80%)	114 (91.2%)		44 (35.2%)	81 (64.8%)	

Of the included respondents, 244 (66.3%) were female and 124 (33.7%) were males, 214 (58.2%) were aged 50-55 years, 187 (50.8%) had a bachelor’s degree, and 24 (6.52%) had completed higher education. A total of 125 (34%) respondents stated that they contracted chickenpox, while 61 (16.6%) did not know if they had ever had chickenpox. Of the respondents, 306 (83.2%) and 190 (51.6%) were aware of the HZ and HZ vaccines, respectively. Awareness regarding HZ was more prevalent among females than males (87.7% vs. 74.2%, respectively, P < 0.05). A statistically significant linear positive trend was observed in the association between education and awareness regarding HZ and its vaccine (P < 0.001). A statistically significant positive association was also observed between prior self-reported chickenpox infection and knowledge regarding HZ and its vaccine (P < 0.001). Saudi respondents were more aware of the HZ vaccine than non-Saudi respondents (53.6% vs. 31.2%, P = 0.026). A statistically significant association was also observed between the city of residency and awareness regarding HZ and its vaccine.

Knowledge regarding HZ and HZ vaccine

Of the respondents, three (0.8%) had good overall knowledge (≥80% of questions answered correctly) about HZ, while 84 (22.8%) had good overall knowledge about the HZ vaccine. A summary of the respondents is presented in the Supplementary Table. The mean knowledge scores (%) regarding HZ and its vaccine were 28.6% and 37.1%, respectively. While 51.6% (n = 190) of the respondents claimed to be aware of the HZ vaccine, only 31.6% (n = 60) had a knowledge score of 80% or above. Three-quarters of the respondents (73.1%, n = 269) could not recognize the link between chickenpox and HZ. The number of respondents who chose “I do not know” ranged from 44.3% to 61.7%. Only 29.3% knew that HZ is not transmitted through close contact, and the same number of respondents knew that there were medications available to treat HZ. Regarding HZ symptoms, 60.3% (n = 222) of the participants correctly identified rash as a clinical feature of HZ. However, other symptoms, such as blisters (39.7%, n = 146) and neuropathic pain (36.4%, n = 134), were less commonly identified by the participants, while blindness and hearing loss were identified by only 8.15% (n = 30) and 5.16% (n = 19), respectively.

Regarding the HZ vaccine, one-third (31.8%, n = 117) of the respondents knew the recommended age group for the HZ vaccine, and only one-quarter (26.1%, n = 96) correctly identified all groups who could take the HZ vaccine. Approximately one-half (48.9%, n = 180) knew that the HZ vaccine could reduce the incidence of the disease.

A positive association was observed between knowledge and education (Table 2). The average knowledge score increased as the level of education increased (P < 0.001). A significant association was observed between age and knowledge regarding HZ, with lower scores observed in respondents aged > 60 years than in respondents under 60 years of age (P < 0.05). Region, employment status, and nationality were not significantly associated with knowledge regarding HZ and its vaccine. The average knowledge score was significantly higher in females than in males (P < 0.05). Prior infection with chickenpox was associated with higher knowledge regarding HZ and its vaccine (P < 0.05).

**Table 2:** Association between knowledge and the respondents’ demographic characteristics HZ: herpes zoster. Analysis was performed using a one-way ANOVA. P-values < 0.05 are deemed statistically significant.

			HZ knowledge		HZ vaccine knowledge	
Variable	Category	N	Mean ± SD	P-value	Mean ± SD	P-value
Age				0.04		0.01
	50-55	214	2.96 ± 2.06		1.96 ± 1.71	
	56–60	97	3.01 ± 2.21		1.98 ± 1.78	
	More than 60	57	2.21 ± 2.23		1.23 ± 1.44	
Aware of HZ				<0.001		<0.001
	No	62	0.77 ± 1.43		0.61 ± 1.23	
	Yes	306	3.28 ± 2.01		2.1 ± 1.68	
Aware of the HZ vaccine				<0.001		<0.001
	No	178	2.09 ± 2.08		1.17 ± 1.54	
	Yes	190	3.58 ± 1.94		2.49 ± 1.61	
Region				0.32		0.12
	Central Region	69	2.52 ± 2.26		1.65 ± 1.83	
	Eastern Region	48	2.54 ± 2.08		1.54 ± 1.58	
	Northern Region	16	3.69 ± 2.06		1.56 ± 1.03	
	Southern Region	66	2.74 ± 1.88		2 ± 1.56	
	Western Region	169	3.05 ± 2.19		1.99 ± 1.79	
Educational level				<0.001		<0.001
	Illiterate	19	0.95 ± 1.61		0.58 ± 1.26	
	Elementary	14	1.79 ± 1.58		1.36 ± 1.82	
	Intermediate	26	2.5 ± 2.39		1.15 ± 1.16	
	Higher education	24	2.96 ± 2.16		2.17 ± 1.81	
	Secondary diploma	98	2.82 ± 2.06		1.53 ± 1.48	
	University degree	187	3.19 ± 2.11		2.25 ± 1.78	
Employment status				0.64		0.64
	Employed	189	2.93 ± 2.19		1.89 ± 1.76	
	Retired	179	2.79 ± 2.08		1.81 ± 1.65	
Gender				0.008		0.008
	Female	244	3.13 ± 2.12		2.02 ± 1.74	
	Male	124	2.33 ± 2.09		1.52 ± 1.59	
Ever had chickenpox				0.006		0.003
	I do not know	61	2.05 ± 2.18		1.28 ± 1.68	
	No	182	2.74 ± 2.18		1.82 ± 1.74	
	Yes	125	3.42 ± 1.91		2.18 ± 1.6	
Nationality				0.18		0.18
	Non-Saudi	32	2.72 ± 2.47		1.47 ± 1.46	
	Saudi	336	2.87 ± 2.11		1.89 ± 1.73	

Based on a multivariate linear regression (Table 3), education was associated with knowledge regarding HZ (B = 0.29, P < 0.001) and its vaccine (B = 0.26, P < 0.001). The non-significant association of nationality, region, and employment status with knowledge did not change. Male gender was associated with lower average knowledge regarding HZ (B = -0.57, P = 0.02) and its vaccine (B = -0.32, P = 0.107), although the latter association was not statistically significant. Older age (>60 years) was associated with lower average knowledge regarding HZ and its vaccine, although the former association was not statistically significant. Prior chickenpox infection was associated with a higher level of knowledge regarding HZ (B = 1.08, P = 0.001) and its vaccine (B = 0.59, P = 0.027).

**Table 3:** Multivariate analysis of the factors associated with HZ and its vaccine HZ: herpes zoster. Analysis was performed using linear regression. P-values < 0.05 are deemed statistically significant.

	HZ knowledge			HZ vaccine knowledge		
Predictors	Estimates	95% CI	P-value	Estimates	95% CI	P-value
Age						
≤60	Reference			Reference		
>60	-0.42	–1.02 to 0.18	0.172	-0.51	–0.99 to –0.03	0.037
Education	0.29	0.13 to 0.45	<0.001	0.26	0.13 to 0.39	<0.001
Gender						
Female	Reference			Reference		
Male	–0.57	–1.05 to –0.09	0.020	–0.32	–0.70 to 0.07	0.107
City						
Central Region	Reference			Reference		
Eastern Region	0.16	–0.61 to 0.93	0.685	0.02	–0.60 to 0.64	0.954
Northern Region	0.99	–0.14 to 2.11	0.085	-0.16	–1.07 to 0.74	0.723
Southern Region	0.08	–0.63 to 0.78	0.835	0.27	–0.30 to 0.84	0.355
Western Region	0.14	–0.46 to 0.74	0.654	0.04	–0.44 to 0.53	0.856
Employment status						
Unemployed	Reference			Reference		
Retired	0.12	–0.33 to 0.58	0.594	0.18	–0.19 to 0.54	0.341
Ever had chickenpox						
I do not know	Reference			Reference		
No	0.56	–0.04 to 1.17	0.067	0.40	–0.09 to 0.89	0.107
Yes	1.08	0.43 to 1.73	0.001	0.59	0.07 to 1.12	0.027
Nationality						
Non-Saudi	Reference			Reference		
Saudi	-0.15	–0.92 to 0.62	0.704	0.13	–0.50 to 0.75	0.686

Attitudes and practices related to HZ vaccination

**Figure 1:**
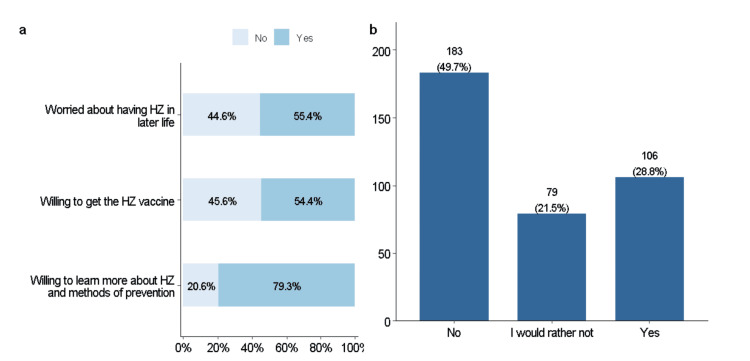
(A) Attitudes and practices related to HZ vaccination and (B) willingness to pay for the HZ vaccine HZ: herpes zoster.

Three-quarters of the respondents were willing to learn more about HZ and prevention methods. Only 55.4% of the respondents were worried about having HZ in later life, and 54.4% were willing to get the HZ vaccine. Only 28.8% of the respondents were willing to pay for the HZ vaccine out of pocket, while 21.5% could afford to pay but would rather not do so. The remaining 49.7% were not willing to pay out of pocket. The participants were aware of the approximate price of the vaccine and understood that the question only referred to the HZ vaccine provided by non-governmental hospitals (Figure 1).

The results showed that worrying about having HZ in later life was significantly associated with the willingness to receive the HZ vaccine (OR = 7.55, P < 0.001). Previous chickenpox infection was also associated with the willingness to receive the HZ vaccine (OR = 2.5, P < 0.05). Similarly, higher education was associated with the odds of receiving the HZ vaccine (OR = 1.2, P = 0.05) (Table 4).

**Table 4:** Factors associated with the willingness to receive the HZ vaccine HZ: herpes zoster. Analysis was performed using binary logistic regression. Worried about having HZ included as an additional covariate. P-values < 0.05 are deemed statistically significant.

Predictors	Odds ratios	95% CI	P-value
Gender: male vs. female	1.23	0.73 – 2.09	0.435
Age: > 60 vs. ≤ 60	1.45	0.73 – 2.89	0.285
Nationality: Saudi vs. non-Saudi	0.82	0.34 – 1.97	0.660
Employment status: retired vs. employed	0.88	0.53 – 1.47	0.637
Education	1.20	1.00 – 1.44	0.050
Worried about having HZ in later life: yes vs. no	7.55	4.62 – 12.34	<0.001
Ever had chickenpox			
I do not know	Reference		
No	0.80	0.41 – 1.57	0.517
Yes	2.50	1.19 – 5.22	0.015

## Discussion

Several studies worldwide have evaluated the impact of the HZ vaccine on at-risk populations and the decreased economic burden due to vaccine administration. However, only two studies have examined knowledge regarding HZ and its vaccine in the Middle East to understand the hurdles to immunization [11,12].

In this study, 83.2% of the respondents were aware of HZ, while only 51.6% were aware of its vaccine. A previous study in the Western Region of Saudi Arabia showed that approximately 50% of the respondents were aware of the HZ vaccine, which is similar to what was reported in this study and suggests a knowledge gap in this area [12]. According to the respondents in that study, a lack of knowledge regarding vaccines was the most common barrier [12]. The findings also align with those from a multicenter study in South Korea, in which nearly half of the participants were aware of the HZ vaccination, while over 80% were aware of the disease [15]. However, our results contrast with a previous study in the UAE, which reported that only 15% of individuals were aware of the HZ vaccination, while just over 60% were aware of HZ [11]. Importantly, a United States (US) study indicated that participants’ motivation to acquire the vaccination was strongly impacted by increasing knowledge about HZ and its vaccine [16].

In the current study, a negative correlation was observed between increasing age and male gender with the willingness to receive the HZ vaccine, while a positive correlation was observed with academic attainment and a history of chickenpox. Similar results have been reported in other studies [10,13,17], emphasizing the need to focus on these subgroups, especially the elderly, who are at a higher risk of HZ. Socioeconomic status (SES) also negatively impacted the respondents’ willingness to vaccinate, similar to what has been reported in China. This can be attributed to the positive correlation between income, education, and SES, which affects the willingness to pay.

Currently, HZ therapy entails the use of antiviral medications and pain management; however, HZ prophylaxis in older persons through vaccination has been strongly advised [18]. Nonetheless, several studies have demonstrated that HZ vaccination rates are exceedingly low in various geographic regions. Around 5% of Saudi Arabians have received the HZ vaccination, while only 3% of individuals in the UAE and Hong Kong and 8% in the US have received it [11,13,16]. In a Chinese study, 81% of the respondents refused to get immunized for HZ [19].

In the current study, only 54.4% of respondents indicated a willingness to vaccinate, similar to the pooled estimate from a systematic review and meta-analysis, which reported an even lower rate of 28.8% [10]. These results may be due to the lack of awareness of the vaccine’s existence, as shown in a previous study conducted in the Western Region of Saudi Arabia^ ^[12]. Similarly, in a study in Italy, 91% of the interviewed respondents were unaware of the HZ vaccine [17]. In Australia, South Korea, and France, vaccination willingness was approximately 89%, 86%, and 68%, respectively, higher than that reported in the current study [10]. In contrast, respondents in China and the United Kingdom (UK) had willingness rates of about 35% [10]. The low willingness to vaccinate may be attributed to poor knowledge about the disease and the vaccine.

Only 0.8% and 22.8% of the respondents had good overall knowledge about HZ and its vaccine, respectively. While 51.6% claimed to be aware of the HZ vaccine, only 31.6% had appropriate knowledge. The results also showed a lack of knowledge regarding the symptoms of HZ, including PHN, which was identified as a symptom of HZ by only one-third of the respondents. PHN is a common adverse outcome of HZ, causing persistent pain, encephalitis, meningitis, and vision-threatening ocular problems after a rash outbreak [11]. Moreover, only 5.16% of the respondents identified hearing loss as a symptom, while 31% were unaware of any of the symptoms.

These results suggest that efforts must be made to raise the Saudi population's awareness regarding the HZ vaccine to enhance individuals’ willingness to vaccinate and enhance the role of health care personnel (HCP), who substantially influence the decision to vaccinate. Results from a meta-analysis suggested that the vaccination willingness rate was 75.2% for individuals who received recommendations from HCP compared to 49.4% for those who did not [10]. In South Korea, a physician’s suggestion was shown to reverse 69.5% of the refusals to accept the HZ vaccine [20]. Other strategies might include counseling sessions in a community pharmacy, which were shown to be effective on a small scale in a US study [21]. In that study, immunization-certified student pharmacists provided patient education on HZ and Zostavax® to unvaccinated patients using the Shingles Vaccine Information Statement [21].

Limitations

As an online questionnaire was used to collect data, the results may not represent the whole population of Saudi Arabia. Moreover, the participants were recruited through convenience sampling, which increases the possibility of selection bias and may impact the generalizability of the results. This study had a higher female than male population along with an unequal distribution of Saudi Arabia regions. Recall bias is another limitation, as the participants were asked to self-report their chickenpox infection history.

## Conclusions

The level of knowledge regarding HZ and its vaccine was low among the target Saudi population. However, a high proportion of the respondents had a willingness to learn about HZ as well as a moderate willingness to vaccinate against HZ. The results suggest that educational campaigns on HZ and the HZ vaccine targeting at-risk groups are needed to raise awareness and expand the public’s knowledge. Additionally, HCP recommendation of the HZ vaccine to the target population should be encouraged, as it is an important factor in vaccine acceptance.

## Appendices

Supplementary table

**Table 5:** Responses to knowledge questions * Correct answer. HZ: herpes zoster.

	All, N = 368	N
	N (%)	
Ever heard about HZ?		368
No	62 (16.8%)	
Yes	306 (83.2%)	
Ever heard about the HZ vaccine?		368
No	178 (48.4%)	
Yes	190 (51.6%)	
An individual is at risk of HZ if he/she has chickenpox		368
Do not know	227 (61.7%)	
FALSE	42 (11.4%)	
TRUE*	99 (26.9%)	
HZ is more prevalent among immunocompromised individuals		368
Do not know	201 (54.6%)	
FALSE	36 (9.78%)	
TRUE*	131 (35.6%)	
HZ only affects people over 50 years of age		368
Do not know	187 (50.8%)	
FALSE*	75 (20.4%)	
TRUE	106 (28.8%)	
Individuals who have come into contact with HZ patients will acquire HZ		368
Do not know	175 (47.6%)	
FALSE*	108 (29.3%)	
TRUE	85 (23.1%)	
There are no drugs available for treating HZ		368
Do not know	183 (49.7%)	
FALSE*	108 (29.3%)	
TRUE	77 (20.9%)	
The HZ vaccine can reduce the incidence of the disease		368
Do not know	163 (44.3%)	
FALSE	25 (6.79%)	
TRUE*	180 (48.9%)	
HZ symptoms		
Rash*	222 (60.3%)	
Blisters*	146 (39.7%)	
Blindness*	30 (8.15%)	
Neuropathic pain*	134 (36.4%)	
Hearing loss*	19 (5.16%)	
Do not know	114 (31.0%)	
The recommended age group for vaccination against HZ		368
Do not know	174 (47.3%)	
Less than 25 years old	20 (5.43%)	
More than 50 years old*	117 (31.8%)	
No age limit	57 (15.5%)	
Which group of people can take the HZ vaccination?		
All the above*	96 (26.1%)	
Did not have/unsure of the history of chickenpox	44 (12.0%)	
Do not know	175 (47.6%)	
Had chickenpox, but no HZ	37 (10.1%)	
Had HZ before	16 (4.35%)	
